# Imaging of early-stage osteoarthritis: the needs and challenges for diagnosis and classification

**DOI:** 10.1007/s00256-023-04355-y

**Published:** 2023-05-08

**Authors:** Edwin H. G. Oei, Jos Runhaar

**Affiliations:** 1 Department of Radiology & Nuclear Medicine, Erasmus MC University Medical Center Rotterdam, PO-Box 2040, 3000 CA Rotterdam, the Netherlands; 2 Department of General Practice, Erasmus MC University Medical Center Rotterdam, PO-Box 2040, 3000 CA Rotterdam, the Netherlands

**Keywords:** Osteoarthritis, Diagnosis, Classification, Early-stage

## Abstract

In an effort to boost the development of new management strategies for OA, there is currently a shift in focus towards the diagnosis and treatment of early-stage OA. It is important to distinguish diagnosis from classification of early-stage OA. Diagnosis takes place in clinical practice, whereas classification is a process to stratify participants with OA in clinical research. For both purposes, there is an important opportunity for imaging, especially with MRI. The needs and challenges differ for early-stage OA diagnosis versus classification. Although it fulfils the need of high sensitivity and specificity for making a correct diagnosis, implementation of MRI in clinical practice is challenged by long acquisition times and high costs. For classification in clinical research, more advanced MRI protocols can be applied, such as quantitative, contrast-enhanced, or hybrid techniques, as well as advanced image analysis methods including 3D morphometric assessments of joint tissues and artificial intelligence approaches. It is necessary to follow a step-wise and structured approach that comprises, technical validation, biological validation, clinical validation, qualification, and cost-effectiveness, before new imaging biomarkers can be implemented in clinical practice or clinical research.

## The early-stage OA concept and its potential

OA is typically diagnosed once pain has become chronic and functional limitations have a severe impact on activities of daily living. Imaging by means of radiography can support the clinical diagnosis of OA by visualizing structural joint abnormalities such as joint space narrowing and osteophyte formation. However, at these advanced stages of OA, radiographic abnormalities are believed to be irreversible. Likewise, when using traditional classification criteria for OA such as the ACR-criteria [1] or radiographic Kellgren and Lawrence (KL) grading [2] for patient enrollment in clinical trials of new therapies, the disease is in such an advanced stage that development of disease modifying treatments for OA has been unsuccessful. In order to force a breakthrough in the management options for OA and reduce the tremendous consequences for patients and the society, there is a widely supported call to shift our focus towards early-stage OA diagnosis and treatment [3–8]. At an early stage, progression of pain severity and transition into chronic pain might be preventable, functional limitations are not yet too severe to interfere with advises to adopt a healthy lifestyle, and progression of structural abnormalities could potentially be halted or even reversed This narrative review paper is aimed at describing the needs and challenges for imaging in the context of early-stage OA. Particularly, we discuss requirements for imaging acquisition and image analysis for the purpose of diagnosis and classification of early-stage OA, and how current imaging methods meet these requirements. It is not the purpose of this article to describe specific imaging techniques in detail, as this will be covered in other chapters of this special issue.

A literature search was conducted in PubMed for relevant articles on the topic of early-stage OA diagnosis and classification, imaging of early-stage OA using various modalities, and combinations of these. Articles were screened and selected by both authors, based on relevance.

## Diagnosis vs. classification of early-stage OA

A fundamental challenge in early-stage OA research is to actually identify those individuals with early-stage OA. In literature, many varying definitions are used, e.g., KL ≤ 2 [9, 10], KL < 2 in combination with clinical symptoms [8], or first-time consulters in primary care [11], but there is currently no established consensus on how to define early-stage OA [12]. It is important to distinguish the diagnosis of early-stage OA from the classification of early-stage OA [13]. Diagnosis takes place in clinical practice while classification is considered a process to stratify participants with OA in clinical research. The fluctuating course of symptoms in early-stage OA makes both the diagnosis and classification of early-stage OA based on clinical symptoms only difficult [12–14]. This could provide an important opportunity for imaging as an adjunct to the clinical diagnosis. However, imaging should ideally be performed at the time of symptoms, which requires feasible imaging tools in terms of accessibility, speed and costs.

## Diagnosis of early-stage OA and the role of imaging

Diagnosis of early-stage OA in clinical practice should identify all subjects with the disease, and should help clinicians to initiate proper treatment strategies. For diagnostic criteria, a high sensitivity and specificity of the criteria are important. A potentially suitable imaging modality that would fulfil these prerequisites is magnetic resonance imaging (MRI). Unlike radiography, MRI is capable of visualizing structural damage in all tissues in and around the joint that are affected by OA [14]. These tissues include the articular cartilage, bone marrow, meniscus, ligaments, synovium, fat pads, and peri-articular musculature, some of which are believed to play an important role in knee OA [15], particularly in the earlier disease stages. Similar to knee OA, MRI is suitable to visualize changes in the soft tissues in and around the hip joint, including hip-specific structures believed to play a role in OA such as the labrum and ligamentum teres [16].

With the advent of novel MRI techniques the sensitivity of MRI can be further increased. Examples of these include so-called quantitative MRI techniques — also referred to as biochemical or compositional MRI techniques — that allow the measurement of important constituents of the articular cartilage [17]. With T2 mapping, it is possible to determine the amount and network integrity of collagen, while other techniques such as T1ρ, GagCEST, and sodium MRI have been shown to be capable of assessing the amount of proteoglycans [18]. These quantitative MRI techniques have been mostly applied to the knee, followed by the hip joint [19, 20]. When a gadolinium contrast agent is administered, it is possible to reliably assess synovitis in the joint [21], and when this is combined with a dynamic contrast-enhanced MRI (DCE-MRI) acquisition, it is possible to quantitatively characterize the perfusion in the synovium [22] and other tissues and lesions that have been linked to inflammatory pathways in OA, such as the infrapatellar fad pad [23] and bone marrow lesions [24].

There are, however, significant drawbacks of MRI that preclude the application in routine patient care for patients with early-stage OA. A typical MRI examination may take approximately 30 min and even longer if advanced, quantitative techniques are added. These long acquisition times are a significant factor to the restricted accessibility — in terms of waiting lists — and high costs of MRI. Hence, a large research effort is currently ongoing to accelerate the speed of MRI acquisitions, with varying approaches including new rapid MRI pulse sequences [25] and advanced MR image reconstruction techniques such as compressed sensing [26], MR fingerprinting [27] and deep learning reconstruction algorithms [28, 29]. Obviously, the use of contrast agents on a large-scale in a routine clinical setting would be undesirable. Several studies have been performed on MRI of synovitis without the use of contrast agents, indicating that synovitis could be detected, but was underestimated in terms of severity compared to contrast-enhanced MRI as the reference standard [30].

As an alternative to MRI, ultrasound has also been applied to assess OA features, especially in the knees and hands [31], although most studies have not specifically evaluated the role of ultrasound in early-stage OA. These studies have shown that ultrasound seems to be particularly capable of assessing osteophytes [32], synovitis [33], meniscus extrusion [34] as features of knee OA, and a few studies have even shown its capability to assess portions of the articular cartilage of the knee [35]. In the hands, ultrasound has been mainly used to assess inflammatory features of OA using combinations of gray-scale and power Doppler acquisitions. Studies have shown that inflammatory features on ultrasound such as joint effusion, synovial thickening and increased Power Doppler signal at earlier stages of hand OA are associated with progression [36, 37]. Advantages of ultrasound include the high spatial resolution when high-frequency transducers are applied, the low costs, and wide availability, increasingly in primary care settings too. Drawbacks are the incapability of ultrasound to assess intra-articular structures and operator dependency, although an excellent inter-observer agreement for knee OA assessment was reported in several studies [32, 38].

## Classification of early-stage OA and the role of imaging

Classification of early-stage OA aims for identifying a homogeneous group of individuals with early-stage OA for enrolment in clinical trials. For classification criteria, a high specificity is required, while a lower sensitivity is allowed [4, 13]. Although the prerequisites differ for diagnostic versus classification criteria, MRI seems to be suitable to serve both roles. MRI is both sensitive to features related to OA, and specific in the way that several combinations of features, such as non-focal cartilage loss, BMLs, and osteophytes, have been clearly linked to OA. Nevertheless, many clinical trials still enroll subjects based on radiographic imaging alone. For a research setting, more advanced imaging modalities and imaging analysis techniques could be feasible for identifying individuals with early-stage knee OA, as in general more time and more financial resources are available for patient stratification. Therefore, the use of advanced MRI techniques such as quantitative techniques or contrast-enhanced methods mentioned above, is more accepted in the setting of OA patient classification than for diagnosis.

There is also a large ongoing activity in field of image analysis to aid the detection and characterization of early-stage OA. After segmentation of various joint structures, for example the articular cartilage, bones and menisci, their morphometric features (e.g., thickness, area, volume, or shape) can be determined. Change of these measures have been linked to OA onset and progression [39–42]. Here, the segmentation forms a challenge because it is laborious if performed manually, in particular for 3D datasets. To overcome this limitation, a large effort is being undertaken to segment joint tissues automatically, for example with the use of deep learning algorithms [43–45]. Although such advanced image analysis algorithms could provide useful imaging biomarkers for clinical research studies, their applicability as part of diagnostic criteria in patient care remains challenging because they are typically not well integrated in the clinical workflow. Especially for primary care, where diagnostic criteria for early-stage knee OA have the greatest potential, this forms an important barrier [46].

More recently, positron emission tomography–magnetic resonance imaging (PET–MRI) has been proposed as a hybrid technique to visualize metabolic changes in early-stage OA. PET-MRI can be used with different radiotracers such as sodium fluoride to assess bone turnover and fluorodeoxyglucose (FDG) to study glucose metabolism, which is increased with inflammation [47]. PET-MRI is infeasible for large clinical studies, because it is expensive and only available in a limited number of centers, but it has shown promise to increase our understanding about early-stage OA processes and different OA subtypes [48, 49].

## Progression of early-stage OA

A major challenge for the development of criteria for early-stage OA is the lack of a gold standard (or reference standard). For this reason, recent initiatives in the development of diagnostic and classification criteria for early-stage OA have used the progression into ‘established OA’, e.g., KL ≥ 2 or the ACR-criteria for clinical OA, as their reference standard (6′8,) [50]. Until consensus on a gold standard has been established, validating against future OA development is probably the best available option. However, this does suggest that ‘stable early-stage OA’ (early-stage OA without progression) does not exist, which can be debated. Evaluating imaging modalities that can predict the development of established OA among individuals with undifferentiated knee symptoms could identify important features for the diagnosis or classification of early-stage OA. It is important to realize that not all (imaging) features that are theoretically associated with progression into established OA should necessarily be incorporated as part of criteria for early-stage OA, particularly if this would result in the identification of very specific and selected subgroups at risk for progression. This would exclude many cases that should be diagnosed or classified as early-stage OA, limiting the validity of such criteria. Nevertheless, in certain research settings, enriching the population of early-stage OA individuals using such specific (imaging) feature, e.g., if it represents the treatment target for the investigative drug, could be very well justified in light of treatment efficacy and study feasibility.

Due to the slow-developing course of OA, the time between the diagnosis or classification of early-stage OA and the development of established OA can be very long. To overcome this challenge, the evaluation of surrogate outcomes is key and imaging modalities may have the potential to serve as surrogate outcomes in early-stage OA [51, 52]. In the early-stage OA setting, surrogate outcomes should capture changes in a relatively short timeframe after the diagnosis or classification of early-stage OA (e.g., short-term changes in symptoms or short-term structural progression). Importantly, this short-term change should then be related to the long-term development of established OA. Level 1 evidence for surrogate outcomes would come from randomized controlled trials (RCTs), where the intervention effect on the surrogate outcome captures the (entire) effect of the intervention on the development of established OA [53]. The lack of RCTs in early-stage OA hamper this validation strategy and initiating new trials is very time consuming. Level 2 evidence for surrogate outcomes can be obtained from longitudinal cohort studies, where the short-term changes in a potential surrogate outcomes can be evaluated against the long-term development of established OA [53–55].

## Roadmap of imaging biomarkers in early-stage OA

Like any biomarker, defined as ‘ objective indicators of normal biologic processes, pathogenic processes, or pharmacologic responses to therapeutic interventions’ [56, 57], imaging biomarkers must undergo a structured, stepwise, process that includes, among many steps, thorough verification and validation, before they can be applied in clinical care or research. A valuable resource for directions on biomarker development, qualification, and validation is the so-called BIPED model. This model distinguishes Burden of disease, Investigative, Prognostic, Efficacy of intervention, and Diagnostic (BIPED) biomarkers, and provides detailed descriptions for the applicability and validation of such biomarkers [56]. Although the BIPED model was not specifically proposed for imaging biomarkers, if systematically applied to (new) imaging biomarkers, this model can accelerate the applicability of imaging modalities in OA research.

In addition, as there are important differences between biospecimen-derived and imaging biomarkers, specific recommendations have been published for imaging biomarkers [58]. Although developed primarily for other diseases, the proposed processes or tailored ‘roadmaps’ can be equally applied to OA imaging biomarkers. For example, a consensus statement on the roadmap for imaging biomarkers in cancer studies distinguishes the following key aspect of this process: discovery, technical validation, biological validation, clinical validation, qualification, and cost-effectiveness [59]. In the same paper, the authors also make note of two crucial “translational gaps” that must be crossed: imaging biomarkers evaluated in vitro must cross the first gap to become robust medical research tools as a reliable marker to test hypotheses in clinical research, and another gap to be integrated in routine patient care. In reality, for many reasons, bridging these translational gaps has been the bottleneck in the trajectory of many imaging biomarkers, including those for OA.

## Conclusion

In this paper, we have described the needs and challenges for imaging in the context of early-stage OA. It is especially important to distinguish diagnosis of early-stage OA in clinical practice, from classification as a means of stratification in clinical research. Although MRI plays an important role for both purposes, different needs and challenges are involved in diagnosis versus classification. When developing new imaging biomarkers, it is critical to follow a step-wise and structured approach before they can be implemented in clinical practice or clinical research.

## References

[1] Altman R, Asch E, Bloch D, Bole G, Borenstein D, Brandt K, Christy W, Cooke TD, Greenwald R, Hochberg M (1986). Development of criteria for the classification and reporting of osteoarthritis. Classification of osteoarthritis of the knee. Diagnostic and Therapeutic Criteria Committee of the American Rheumatism Association. Arthritis Rheum.

[2] Kellgren JH, Lawrence JS (1957). Radiological assessment of osteo-arthrosis. Ann Rheum Dis.

[3] Hunter DJ, Bierma-Zeinstra S (2019). Osteoarthritis. Lancet.

[4] Mahmoudian A, Lohmander LS, Mobasheri A, Englund M, Luyten FP (2021). Early-stage symptomatic osteoarthritis of the knee - time for action. Nat Rev Rheumatol.

[5] Katz JN, Arant KR, Loeser RF (2021). Diagnosis and treatment of hip and knee osteoarthritis: a review. JAMA.

[6] Runhaar J, Kloppenburg M, Boers M, Bijlsma JWJ, Bierma-Zeinstra SMA, the Ceg (2021). Towards developing diagnostic criteria for early knee osteoarthritis: data from the CHECK study. Rheumatology (Oxford).

[7] Runhaar J, Ozbulut O, Kloppenburg M, Boers M, Bijlsma JWJ, Bierma-Zeinstra SMA, group Ce (2021). Diagnostic criteria for early hip osteoarthritis: first steps, based on the CHECK study. Rheumatology (Oxford).

[8] Luyten FP, Bierma-Zeinstra S, Dell'Accio F, Kraus VB, Nakata K, Sekiya I, Arden NK, Lohmander LS (2018). Toward classification criteria for early osteoarthritis of the knee. Semin Arthritis Rheum.

[9] Madry H, Kon E, Condello V, Peretti GM, Steinwachs M, Seil R, Berruto M, Engebretsen L, Filardo G, Angele P (2016). Early osteoarthritis of the knee. Knee Surg Sports Traumatol Arthrosc.

[10] van Tiel J, Bron EE, Tiderius CJ, Bos PK, Reijman M, Klein S, Verhaar JA, Krestin GP, Weinans H, Kotek G, Oei EH (2013). Reproducibility of 3D delayed gadolinium enhanced MRI of cartilage (dGEMRIC) of the knee at 3.0 T in patients with early stage osteoarthritis. Eur Radiol.

[11] van Berkel AC, Schiphof D, Waarsing JH, Runhaar J, van Ochten JM, Bindels PJE, Bierma-Zeinstra SMA (2021). 10-Year natural course of early hip osteoarthritis in middle-aged persons with hip pain: a CHECK study. Ann Rheum Dis.

[12] Liew JW, King LK, Mahmoudian A, Wang Q, Atkinson HF, Flynn DB, Appleton T, Englund M, Haugen IK, Lohmander LS, Runhaar J, Neogi T, Hawker G. A scoping review of how early-stage knee osteoarthritis has been defined. Osteoarthr Cartil. 2023. e-pub ahead of print. 10.1016/j.joca.2023.04.015 PMC10528892 37225053

[13] Aggarwal R, Ringold S, Khanna D, Neogi T, Johnson SR, Miller A, Brunner HI, Ogawa R, Felson D, Ogdie A, Aletaha D, Feldman BM (2015). Distinctions between diagnostic and classification criteria?. Arthritis Care Res (Hoboken).

[14] Roemer FW, Guermazi A, Demehri S, Wirth W, Kijowski R (2022). Imaging in Osteoarthritis. Osteoarthr Cartil.

[15] Hunter DJ, Guermazi A, Lo GH, Grainger AJ, Conaghan PG, Boudreau RM, Roemer FW (2011). Evolution of semi-quantitative whole joint assessment of knee OA: MOAKS (MRI Osteoarthritis Knee Score). Osteoarthr Cartil.

[16] Lee S, Nardo L, Kumar D, Wyatt CR, Souza RB, Lynch J, McCulloch CE, Majumdar S, Lane NE, Link TM (2015). Scoring hip osteoarthritis with MRI (SHOMRI): a whole joint osteoarthritis evaluation system. J Magn Reson Imaging.

[17] MacKay JW, Low SBL, Smith TO, Toms AP, McCaskie AW, Gilbert FJ (2018). Systematic review and meta-analysis of the reliability and discriminative validity of cartilage compositional MRI in knee osteoarthritis. Osteoarthr Cartil.

[18] Oei EH, van Tiel J, Robinson WH, Gold GE (2014). Quantitative radiologic imaging techniques for articular cartilage composition: toward early diagnosis and development of disease-modifying therapeutics for osteoarthritis. Arthritis Care Res (Hoboken).

[19] Jones CE, Cibere J, Qian H, Zhang H, Guo Y, Russell D, Forster BB, Wong H, Esdaile JM, Wilson DR (2022). Delayed gadolinium-enhanced magnetic resonance imaging of cartilage values in hips with bone marrow lesions. Arthritis Care Res (Hoboken).

[20] Mills ES, Becerra JA, Yensen K, Bolia IK, Shontz EC, Kebaish KJ, Dobitsch A, Hasan LK, Haratian A, Ong CD, Gross J, Petrigliano FA, Weber AE (2022). Current and future advanced imaging modalities for the diagnosis of early osteoarthritis of the hip. Orthop Res Rev.

[21] Guermazi A, Roemer FW, Hayashi D, Crema MD, Niu J, Zhang Y, Marra MD, Katur A, Lynch JA, El-Khoury GY, Baker K, Hughes LB, Nevitt MC, Felson DT (2011). Assessment of synovitis with contrast-enhanced MRI using a whole-joint semiquantitative scoring system in people with, or at high risk of, knee osteoarthritis: the MOST study. Ann Rheum Dis.

[22] MacKay JW, Nezhad FS, Rifai T, Kaggie JD, Naish JH, Roberts C, Graves MJ, Waterton JC, Janiczek RL, Roberts AR, McCaskie A, Gilbert FJ, Parker GJM (2021). Dynamic contrast-enhanced MRI of synovitis in knee osteoarthritis: repeatability, discrimination and sensitivity to change in a prospective experimental study. Eur Radiol.

[23] de Vries BA, van der Heijden RA, Poot DHJ, van Middelkoop M, Meuffels DE, Krestin GP, Oei EHG (2020). Quantitative DCE-MRI demonstrates increased blood perfusion in Hoffa's fat pad signal abnormalities in knee osteoarthritis, but not in patellofemoral pain. Eur Radiol.

[24] de Vries BA, van der Heijden RA, Verschueren J, Bos PK, Poot DHJ, van Tiel J, Kotek G, Krestin GP, Oei EHG (2020). Quantitative subchondral bone perfusion imaging in knee osteoarthritis using dynamic contrast enhanced MRI. Semin Arthritis Rheum.

[25] Eijgenraam SM, Chaudhari AS, Reijman M, Bierma-Zeinstra SMA, Hargreaves BA, Runhaar J, Heijboer FWJ, Gold GE, Oei EHG (2020). Time-saving opportunities in knee osteoarthritis: T(2) mapping and structural imaging of the knee using a single 5-min MRI scan. Eur Radiol.

[26] Kijowski R, Rosas H, Samsonov A, King K, Peters R, Liu F (2017). Knee imaging: rapid three-dimensional fast spin-echo using compressed sensing. J Magn Reson Imaging.

[27] Sharafi A, Zibetti MVW, Chang G, Cloos M, Regatte RR (2022). 3D magnetic resonance fingerprinting for rapid simultaneous T1, T2, and T1rho volumetric mapping of human articular cartilage at 3 T. NMR Biomed.

[28] Herrmann J, Keller G, Gassenmaier S, Nickel D, Koerzdoerfer G, Mostapha M, Almansour H, Afat S, Othman AE (2022). Feasibility of an accelerated 2D-multi-contrast knee MRI protocol using deep-learning image reconstruction: a prospective intraindividual comparison with a standard MRI protocol. Eur Radiol.

[29] Recht MP, Zbontar J, Sodickson DK, Knoll F, Yakubova N, Sriram A, Murrell T, Defazio A, Rabbat M, Rybak L, Kline M, Ciavarra G, Alaia EF, Samim M, Walter WR, Lin DJ, Lui YW, Muckley M, Huang Z, Johnson P, Stern R, Zitnick CL (2020). Using Deep Learning to Accelerate Knee MRI at 3 T: Results of an interchangeability study. AJR Am J Roentgenol.

[30] de Vries BA, Breda SJ, Sveinsson B, McWalter EJ, Meuffels DE, Krestin GP, Hargreaves BA, Gold GE, Oei EHG (2021). Detection of knee synovitis using non-contrast-enhanced qDESS compared with contrast-enhanced MRI. Arthritis Res Ther.

[31] Nevalainen MT, Uusimaa AP, Saarakkala S. The ultrasound assessment of osteoarthritis: the current status. Skeletal Radiol. 2023. 10.1007/s00256-023-04342-3. 10.1007/s00256-023-04342-3 PMC10509065 37060461

[32] Oo WM, Linklater JM, Bennell KL, Daniel MS, Pryke D, Wang X, Yu SP, Deveza L, Duong V, Hunter DJ (2022). Reliability and convergent construct validity of quantitative ultrasound for synovitis, meniscal extrusion, and osteophyte in knee osteoarthritis with MRI. J Ultrasound Med.

[33] de Vries BA, Breda SJ, Meuffels DE, Hanff DF, Hunink MGM, Krestin GP, Oei EHG (2020). Diagnostic accuracy of grayscale, power Doppler and contrast-enhanced ultrasound compared with contrast-enhanced MRI in the visualization of synovitis in knee osteoarthritis. Eur J Radiol.

[34] Kawaguchi K, Enokida M, Otsuki R, Teshima R (2012). Ultrasonographic evaluation of medial radial displacement of the medial meniscus in knee osteoarthritis. Arthritis Rheum.

[35] Kauppinen K, Casula V, Zbyn S, Blanco Sequeiros R, Saarakkala SS, Nevalainen MT (2021). Ultrasonographic assessment of the normal femoral articular cartilage of the knee joint: comparison with 3D MRI. Sci World J.

[36] Mathiessen A, Slatkowsky-Christensen B, Kvien TK, Hammer HB, Haugen IK (2016). Ultrasound-detected inflammation predicts radiographic progression in hand osteoarthritis after 5 years. Ann Rheum Dis.

[37] Kortekaas MC, Kwok WY, Reijnierse M, Kloppenburg M (2015). Inflammatory ultrasound features show independent associations with progression of structural damage after over 2 years of follow-up in patients with hand osteoarthritis. Ann Rheum Dis.

[38] Razek AA, El-Basyouni SR (2016). Ultrasound of knee osteoarthritis: interobserver agreement and correlation with Western Ontario and McMaster Universities Osteoarthritis. Clin Rheumatol.

[39] Xu D, van der Voet J, Hansson NM, Klein S, Oei EHG, Wagner F, Bierma-Zeinstra SMA, Runhaar J (2021). Association between meniscal volume and development of knee osteoarthritis. Rheumatology (Oxford).

[40] Oei EHG, van Zadelhoff TA, Eijgenraam SM, Klein S, Hirvasniemi J, van der Heijden RA (2021). 3D MRI in Osteoarthritis. Semin Musculoskelet Radiol.

[41] Bowes MA, Kacena K, Alabas OA, Brett AD, Dube B, Bodick N, Conaghan PG (2021). Machine-learning, MRI bone shape and important clinical outcomes in osteoarthritis: data from the Osteoarthritis Initiative. Ann Rheum Dis.

[42] Xu D, van der Voet J, Waarsing JH, Oei EH, Klein S, Englund M, Zhang F, Bierma-Zeinstra S, Runhaar J (2021). Are changes in meniscus volume and extrusion associated to knee osteoarthritis development? A structural equation model. Osteoarthr Cartil.

[43] Joseph GB, McCulloch CE, Sohn JH, Pedoia V, Majumdar S, Link TM (2022). AI MSK clinical applications: cartilage and osteoarthritis. Skeletal Radiol.

[44] Gaj S, Yang M, Nakamura K, Li X (2020). Automated cartilage and meniscus segmentation of knee MRI with conditional generative adversarial networks. Magn Reson Med.

[45] Desai AD, Caliva F, Iriondo C, Mortazi A, Jambawalikar S, Bagci U, Perslev M, Igel C, Dam EB, Gaj S, Yang M, Li X, Deniz CM, Juras V, Regatte R, Gold GE, Hargreaves BA, Pedoia V, Chaudhari AS (2021). The International Workshop on Osteoarthritis Imaging Knee MRI Segmentation Challenge: a multi-institute evaluation and analysis framework on a standardized dataset. Radiol Artif Intell.

[46] Wang Q, Runhaar J, Kloppenburg M, Boers M, Bijlsma JWJ, Bierma-Zeinstra SMA, group Ce (2022). Diagnosis for early stage knee osteoarthritis: probability stratification, internal and external validation; data from the CHECK and OAI cohorts. Semin Arthritis Rheum.

[47] Kogan F, Fan AP, McWalter EJ, Oei EHG, Quon A, Gold GE (2017). PET/MRI of metabolic activity in osteoarthritis: a feasibility study. J Magn Reson Imaging.

[48] Watkins LE, Haddock B, MacKay JW, Baker J, Uhlrich SD, Mazzoli V, Gold GE, Kogan F (2022). [(18)F]Sodium fluoride PET-MRI detects increased metabolic bone response to whole-joint loading stress in osteoarthritic knees. Osteoarthr Cartil.

[49] Tibrewala R, Pedoia V, Bucknor M, Majumdar S (2020). Principal component analysis of simultaneous PET-MRI reveals patterns of bone-cartilage interactions in osteoarthritis. J Magn Reson Imaging.

[50] Mahmoudian A, Lohmander LS, Jafari H, Luyten FP (2021). Towards classification criteria for early-stage knee osteoarthritis: a population-based study to enrich for progressors. Semin Arthritis Rheum.

[51] Runhaar J, Bierma-Zeinstra SMA (2022). The challenges in the primary prevention of osteoarthritis. Clin Geriatr Med.

[52] Runhaar J, van Middelkoop M, Oei EHG, Bierma-Zeinstra SMA (2023). Potential surrogate outcomes in individuals at high risk for incident knee osteoarthritis. Osteoarthr Cartil.

[53] Ciani O, Buyse M, Drummond M, Rasi G, Saad ED, Taylor RS (2017). Time to review the role of surrogate end points in health policy: state of the art and the way forward. Value Health.

[54] Katz JN, Collins JE, Jones M, Spindler KP, Marx RG, Mandl LA, Levy BA, Wright R, Jarraya M, Guermazi A, MacFarlane LA, Losina E, Chang Y (2023). Association between structural change over eighteen months and subsequent symptom change in middle-aged patients treated for meniscal tear. Arthritis Care Res (Hoboken).

[55] Runhaar J, Dam EB, Oei EHG, Bierma-Zeinstra SMA (2021). Medial cartilage surface integrity as a surrogate measure for incident radiographic knee osteoarthritis following weight changes. Cartilage.

[56] Bauer DC, Hunter DJ, Abramson SB, Attur M, Corr M, Felson D, Heinegard D, Jordan JM, Kepler TB, Lane NE, Saxne T, Tyree B, Kraus VB, Osteoarthritis BN (2006). Classification of osteoarthritis biomarkers: a proposed approach. Osteoarthr Cartil.

[57] Biomarkers Definitions Working G (2001). Biomarkers and surrogate endpoints: preferred definitions and conceptual framework. Clin Pharmacol Ther.

[58] European Society of R (2013). ESR statement on the stepwise development of imaging biomarkers. Insights Imaging.

[59] O'Connor JP, Aboagye EO, Adams JE, Aerts HJ, Barrington SF, Beer AJ, Boellaard R, Bohndiek SE, Brady M, Brown G, Buckley DL, Chenevert TL, Clarke LP, Collette S, Cook GJ, deSouza NM, Dickson JC, Dive C, Evelhoch JL, Faivre-Finn C, Gallagher FA, Gilbert FJ, Gillies RJ, Goh V, Griffiths JR, Groves AM, Halligan S, Harris AL, Hawkes DJ, Hoekstra OS, Huang EP, Hutton BF, Jackson EF, Jayson GC, Jones A, Koh DM, Lacombe D, Lambin P, Lassau N, Leach MO, Lee TY, Leen EL, Lewis JS, Liu Y, Lythgoe MF, Manoharan P, Maxwell RJ, Miles KA, Morgan B, Morris S, Ng T, Padhani AR, Parker GJ, Partridge M, Pathak AP, Peet AC, Punwani S, Reynolds AR, Robinson SP, Shankar LK, Sharma RA, Soloviev D, Stroobants S, Sullivan DC, Taylor SA, Tofts PS, Tozer GM, van Herk M, Walker-Samuel S, Wason J, Williams KJ, Workman P, Yankeelov TE, Brindle KM, McShane LM, Jackson A, Waterton JC (2017). Imaging biomarker roadmap for cancer studies. Nat Rev Clin Oncol.

